# Cocaine-Induced Delayed Myocardial Infarction Complicated by Apical Thrombus

**DOI:** 10.14740/jocmr2412w

**Published:** 2015-12-03

**Authors:** Rafay Khan, Sabrina Arshed, Waqas Jehangir, Shuvendu Sen, Abdalla Yousif

**Affiliations:** a Department of Internal Medicine, Raritan Bay Medical Center, 530 New Brunswick Avenue, Perth Amboy, NJ 08861, USA

**Keywords:** Acute coronary syndrome, Cocaine, Thrombus, Myocardial infarction

## Abstract

It is well demonstrated in the literature that cocaine use has been well linked to the formation of various forms of acute and chronic cardiovascular problems including but not limited to acute coronary syndromes. However, cocaine has been commonly associated with coronary vasospasms and less commonly with myocardial infarction and the formation of atrial thrombus. Through this case presentation, we illustrate the findings of a 35-year-old gentleman with history of cocaine use presenting with acute coronary syndrome and complicated by thrombus formation. Furthermore, through this report, we illustrate in a patient with no other risk factors and at a young age, how chronic cocaine use or even a history of usage may result in complications even weeks after its consumption.

## Introduction

The acute use of cocaine has been demonstrated in the literature to result in coronary vasospasms but has not been well documented to result in thrombus formation and acute coronary syndrome (ACS) weeks after its effects have worn off. Coleman et al were the first to mention a relationship between ACS and cocaine use [1, 2]. However, it has been reported that the risk of ACS is increased 24-fold during the first hour after cocaine use in patients with normal coronary arteries [1, 3], thus making this case a very unusual presentation of ACS as a result of a history of cocaine usage. It has been researched that the use of cocaine can result in vasoconstriction in patients but can also result in increased platelet activation and aggregation resulting in thrombus formation [1, 4]. Whether this thrombus formation is limited to the coronary arteries or in combination with hypokinesis can increase the risk of atrial thrombus has also not been well documented in the literature.

## Case Report

A 35-year-old male with past medical history of cocaine abuse presented to the emergency room with complaints of sudden chest pain, gradually worsening, and radiating to the left arm. The chest pain began around 3 am and due to its progression with slight shortness of breath, he was advised by his sister to come to the hospital. The patient has no significant family history and social history is significant for smoking one pack per day for the past 8 years, social alcohol use, and occasional cocaine use. However, he last used cocaine 2 weeks prior to admission.

**Figure 1:**
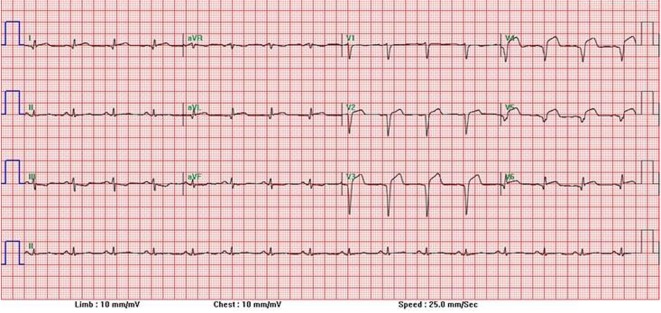
EKG with ST elevations in anterior leads.

Vital signs upon presentation were a blood pressure of 134/86 mm Hg, respiratory rate of 20/min, heart rate of 89 beats/min, temperature of 98 °F, and he was saturating 98% on a non-rebreather. Physical examination otherwise was unremarkable. Laboratory investigation demonstrated a hemoglobin of 14.5, hematocrit of 44.8, leukocytosis of 13.6, and platelets of 330. Basic metabolic panel showed a sodium of 138, potassium of 3.4, chloride of 100, bicarbonate of 26, blood urea nitrogen of 111, creatinine of 1.1, and glucose of 135. Initial arterial blood gas showed a pH of 7.33, pCO_2_ of 36, pO_2_ of 61, and bicarbonate of 91. Urine drug screen was negative. Cardiac enzymes and troponins were unremarkable. Initial electrocardiogram (EKG) demonstrated normal sinus rhythm but early repolarization signs at 4:30 am. Due to the continuous nature of his chest pain, a repeat EKG at 5:30 am was conducted demonstrating ST elevations in the anterior leads consisting of V1-V4 (Fig. 1). Due to his history of cocaine use, acute cardiac intervention was initially withheld and the patient was managed medically and monitored in the intensive care unit.

**Figure 2:**
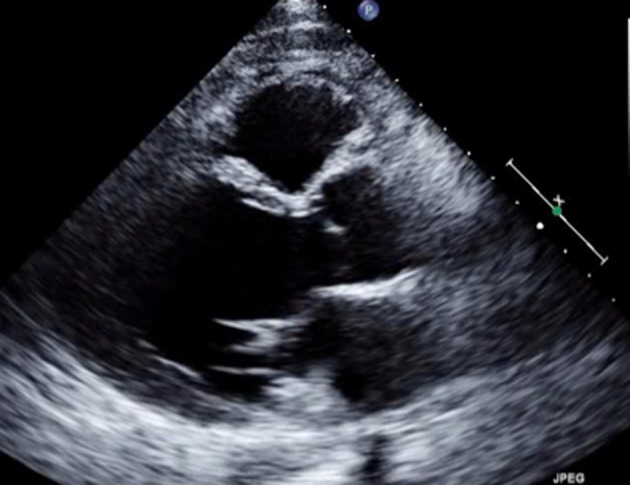
Echocardiogram with severe hypokinesis of anterior, anteroseptal walls, and apex.

The following day, repeat EKG was concerning for ongoing ST elevations and as the urine drug screen was negative, the patient was taken for cardiac catheterization. Cardiac catheterization revealed 100% stenosis of the left anterior descending artery (LAD) with a left ventricle ejection fraction (LVEF) of 30%. Patient underwent emergent percutaneous intervention (PCI) to the LAD stenosis. Follow-up echocardiogram showed an LVEF of 25-30% with severe hypokinesis of the entire anterior, anteroseptal walls, and apex with moderate hypokinesis of the anterolateral wall (Fig. 2). An apical thrombus was found to be highly suspected on the imaging study and the patient was initiated on warfarin. The patient was continued on medical management and safely discharged home with close outpatient monitoring with his cardiologist. He would be scheduled for a repeat echocardiogram in 6 months for further evaluation and intervention.

## Discussion

Greater than 20% of patients who went to the hospital due to overconsumption of cocaine had symptoms related to the cardiovascular system [4]. These symptoms can be a result of ACS, arrhythmia, aortic dissection, endocarditis, deep venous thrombosis, or even aneurysm rupture [5]. Most cases reporting arterial thrombosis are rare as a result of cocaine, however when present, usually consist of small arteries such as the cerebral or coronary arteries [5]. Moreover, it is unusual to find cocaine causing effects of thrombosis over 2 weeks after its consumption as it is most commonly associated with complications within the first 24 h. It is even rarer to see its effects resulting in thrombus formation either directly or indirectly via hypokinesis from ACS. This train of thought however still requires considerable research.

The exact mechanism by which cocaine results in arterial thrombosis remains poorly studied. It has been linked to many cardiovascular adverse effects that may be largely a result of central and peripheral adrenergic stimulation that may influence arterial circulation [4]. It results in inhibition of the reuptake of both norepinephrine and dopamine at sympathetic preganglionic nerve endings that result in catecholamine effects, in turn causing vasoconstriction of the arteries and hypertension. This may result in a significant decrease in coronary blood flow, luminal diameter, and thus increase the chance of myocardial infarction. Another postulated method of action well described is the result of increased platelet aggregation and release of vasoactive mediators which themselves may increase arterial thrombosis [6]. A study conducted by Kugelmass et al had previously demonstrated that intravenous cocaine administration can activate circulating platelets via increased expression of P-selectin found on surfaces of activated platelets [5, 7]. Whether this mechanism can also increase the risk of atrial thrombus formation along with the underlying hypokinesis still needs further analysis as demonstrated by our case presentation.

In terms of thrombus formation and ACS, there has been a correlation between the two. However, no real association has been demonstrated with rapid formation of thrombus formation a few weeks after cocaine use in a patient with otherwise no other risk factors. In terms of managing these patients post-intervention, the choice of treatment requires a combination of therapies bearing in mind risk of bleeding, patient’s age, and many other risk factors. The current consensus in patients with mural thrombi is the use of an oral anticoagulant therapy with a vitamin K antagonist for up to 6 months [8]. This consensus however has yet to be revised since the implementation of stenting and dual anti-platelet therapy. The optimum duration of this triple antithrombotic regimen also remains unknown and still requires further research. In some analysis, if imaging 3 months into the therapy demonstrates resolution of the thrombus then anticoagulation therapy may be discontinued especially if there is recovery of apical wall motion [9]. Therefore, there are overall three factors which have been implicated in the pathogenesis of cocaine-induced ACS: increased oxygen demand of the heart, vasoconstriction of the coronary arteries, and increased platelet aggregation [4, 7]. On the other hand, physicians need to remain cautious when managing patients with cocaine-related symptoms as a delay in treatment can be detrimental and should thus not base their choice of intervention on one risk factor but as the overall clinical presentation of the patient.

### Conclusion

Physicians should maintain a high index of suspicion when treating patients with a history of cocaine use; however, it is important to be weary of the timeline of its consumption. Through this case, it is apparent that cocaine can in fact have cardiovascular complications up to weeks after last consumption. Whether it directly can result in thrombus formation or is secondary to ACS remains unknown. However, it provides physicians an example of a case that vasospasm and acute chest pain from cocaine consumption may not be a simple case as initially perceived.

## References

[1] Apostolakis E, Tsigkas G, Baikoussis NG, Koniari I, Alexopoulos D (2010). Acute left main coronary artery thrombosis due to cocaine use. J Cardiothorac Surg.

[2] Coleman DL, Ross TF, Naughton JL (1982). Myocardial ischemia and infarction related to recreational cocaine use. West J Med.

[3] Mittleman MA, Mintzer D, Maclure M, Tofler GH, Sherwood JB, Muller JE (1999). Triggering of myocardial infarction by cocaine. Circulation.

[4] Lange RA, Hillis LD (2001). Cardiovascular complications of cocaine use. N Engl J Med.

[5] Zhou W, Lin PH, Bush RL, Nguyen L, Lumsden AB (2004). Acute arterial thrombosis associated with cocaine abuse. J Vasc Surg.

[6] Togna G, Tempesta E, Togna AR, Dolci N, Cebo B, Caprino L (1985). Platelet responsiveness and biosynthesis of thromboxane and prostacyclin in response to in vitro cocaine treatment. Haemostasis.

[7] Kugelmass AD, Oda A, Monahan K, Cabral C, Ware JA (1993). Activation of human platelets by cocaine. Circulation.

[8] Lacalzada J, Mari B, Izquierdo MM, Sanchez-Grande A, de la Rosa A, Laynez I (2013). Recurrent intraventricular thrombus six months after ST-elevation myocardial infarction in a diabetic man: a case report. BMC Res Notes.

[9] Douglas PS, Garcia MJ, Haines DE, Lai WW, Manning WJ, Patel AR, Picard MH (2011). ACCF/ASE/AHA/ASNC/HFSA/HRS/SCAI/SCCM/SCCT/SCMR 2011 Appropriate Use Criteria for Echocardiography. A Report of the American College of Cardiology Foundation Appropriate Use Criteria Task Force, American Society of Echocardiography, American Heart Association, American Society of Nuclear Cardiology, Heart Failure Society of America, Heart Rhythm Society, Society for Cardiovascular Angiography and Interventions, Society of Critical Care Medicine, Society of Cardiovascular Computed Tomography, and Society for Cardiovascular Magnetic Resonance Endorsed by the American College of Chest Physicians. J Am Coll Cardiol.

